# Supplementary material for: Potential impacts of Washington State’s wildfire worker protection rule on construction workers
Source: Ann Work Expo Health. 2021 Dec 22;66(4):419–32. doi: 10.1093/annweh/wxab115 (PMC9030230; doi:10.1093/annweh/wxab115)
Supplement: wxab115_suppl_Supplementary_Material [file wxab115_suppl_supplementary_material.docx]

**Potential impacts of Washington State’s wildfire worker protection rule on construction workers**

Christopher Zuidema^1^, Elena Austin^1^, Martin A. Cohen^1^, Edward Kasner^1^, Lilian Liu^1^, Tania Busch Isaksen^1^, Ken-Yu Lin^2^, June Spector^1, 3^, Edmund Seto^1, *^

^1^ Department of Environmental and Occupational Health Sciences, University of Washington, Seattle, WA, USA

^2^ Department of Construction Management, University of Washington, Seattle, WA, USA

^3^ Department of Medicine, University of Washington, Seattle, WA, USA

^*^ Corresponding author

# **SUPPLEMENTAL MATERIALS**


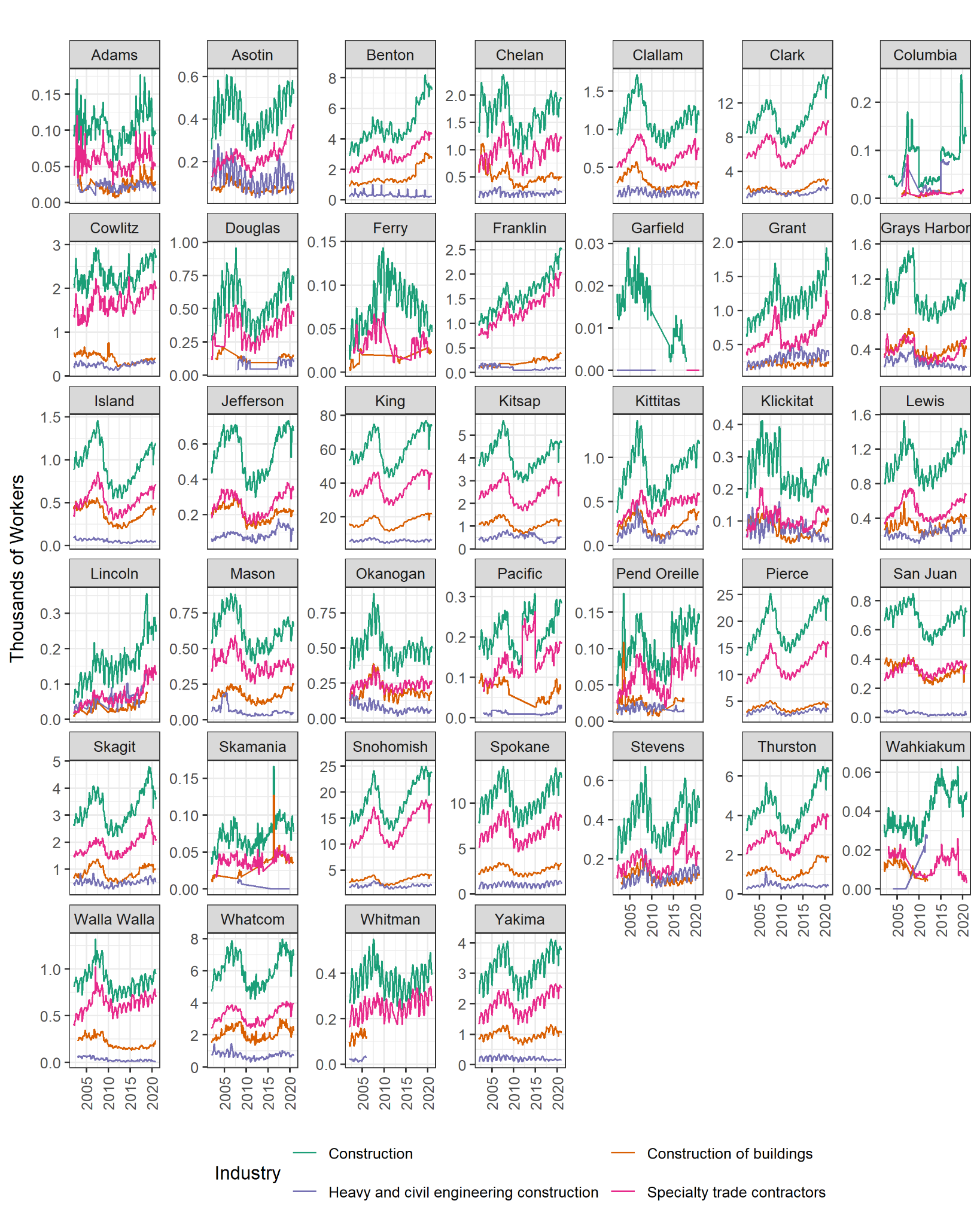


**Figure S1.** Monthly counts of WA construction workers.


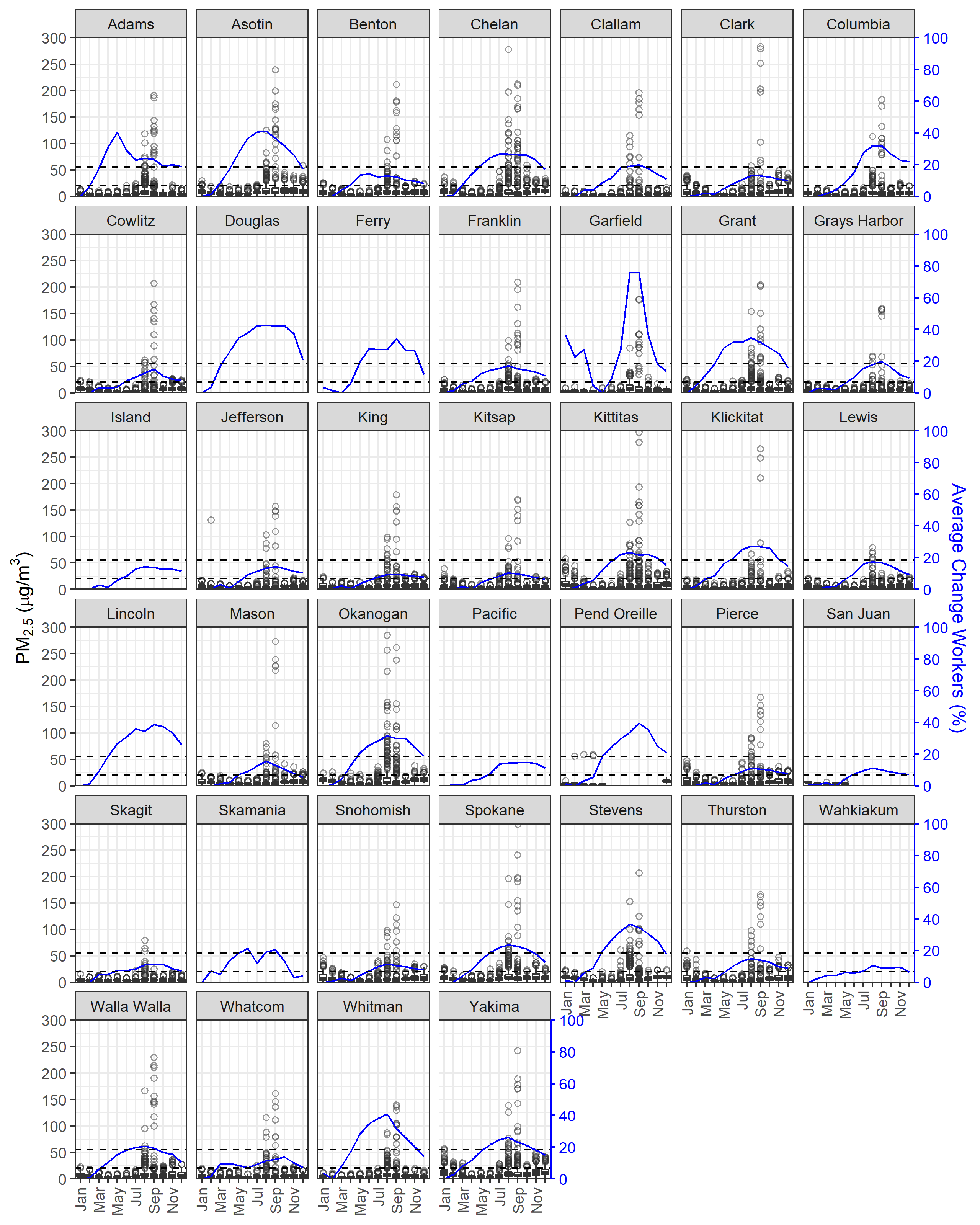


**Figure S2.** Mean daily PM_2.5_ concentration and average monthly percent difference in construction workers from the month with the lowest number of workers for all WA counties; 2011-2020. (Note: axes were restricted, omitting outlying data points above 300 µg/m^3^.


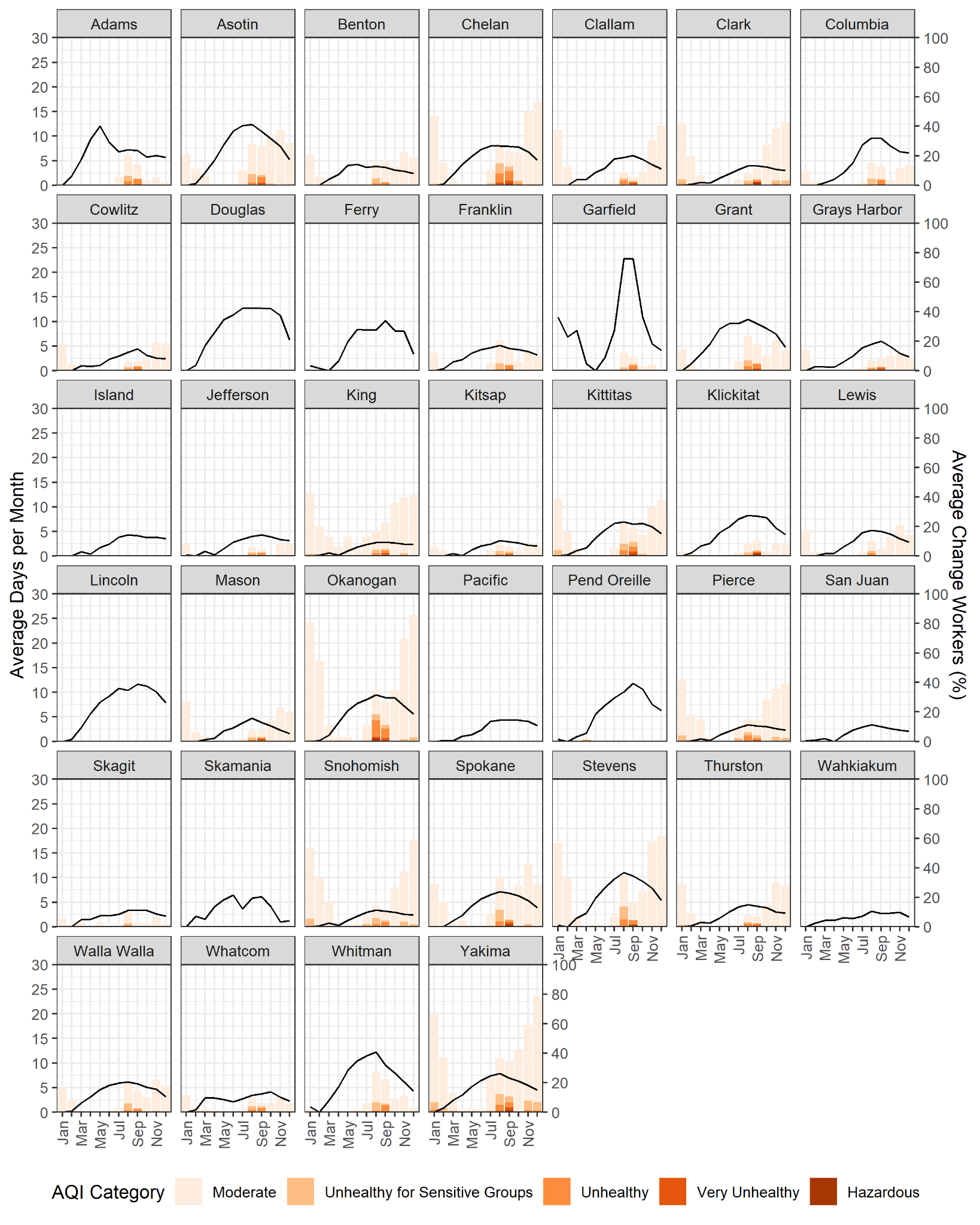


**Figure S3.** Average number of days per month with AQI worse than “good” and average monthly percent difference in construction workers from the month with the lowest number of workers averaged over 2011-2020 for all WA counties.


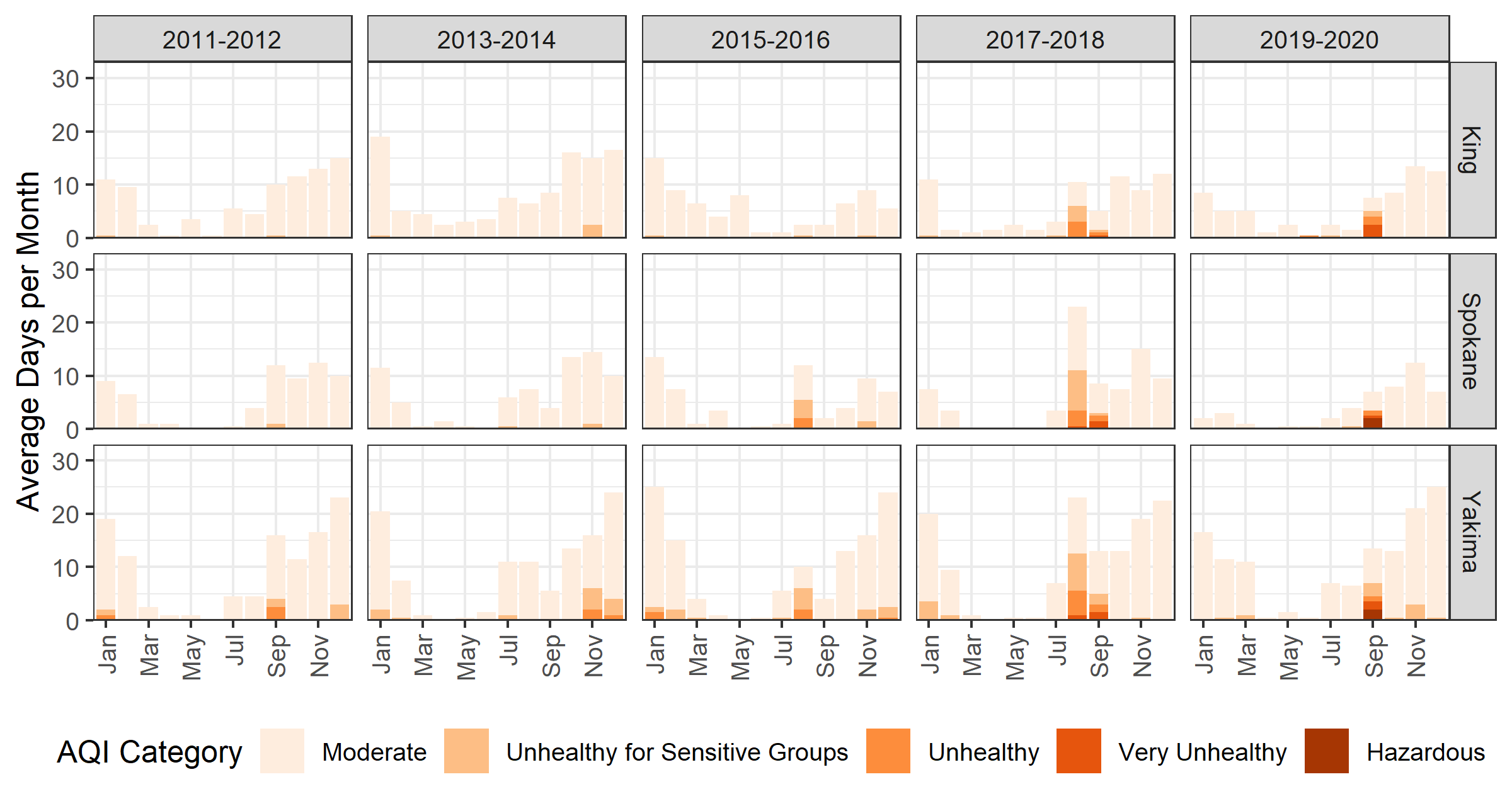


**Figure S4:** Average number of days per month with AQI warnings by county by month for 2-year periods; 2011-2020.

**Table S1.** Summary of AQI and WAQA levels and corresponding PM_2.5_ concentrations.

| **Level of Concern** | **Index Value**  **(AQI & WAQA)** | **EPA PM_2.5_ (µg/m^3^) ^a^** | **WAQA PM_2.5_ (µg/m^3^) ^a^** | **Air Quality Description** |
| --- | --- | --- | --- | --- |
| Good | 0 – 50 | 0.0 – 12.0 | 0.0 – 12.0 | Air quality is satisfactory, and air pollution poses little or no risk. |
| Moderate | 51 – 100 | 12.1 – 35.4 | 12.1 – 20.4 | Air quality is acceptable. However, there may be a risk for some people, particularly those who are unusually sensitive to air pollution. |
| Unhealthy for  Sensitive Groups | 101 – 150 | 35.5 – 55.4 | 20.5 – 35.4 | Members of sensitive groups may experience health effects. The general public is less likely to be affected. |
| Unhealthy | 151 – 200 | 55.5 – 150.4 | 35.5 – 80.4 | Some members of the general public may experience health effects; members of sensitive groups may experience more serious health effects. |
| Very Unhealthy | 201 – 300 | 150.5 – 250.4 | 80.5 – 150.4 | Health alert: The risk of health effects is increased for everyone. |
| Hazardous | ≥301 | ≥250.5 | ≥150.5 | Health warning of emergency conditions: everyone is more likely to be affected. |

^a^ 24-hr average concentration

**Table S2.** Summary of the number of construction workers by NAICS code in WA for 2020.

| **NAICS code** | | | **Industry** | **Potential for Outdoor Work** | **Firms** | **Workers** | **Percent of 2-digit NAICS** | **Percent of 3-digit NAICS** |
| --- | --- | --- | --- | --- | --- | --- | --- | --- |
| 23 |  |  | Construction |  | 26977 | 199784 | 100.0 |  |
|  | 236 |  | Construction of buildings |  | 9478 | 51636 | 25.8 | 100.0 |
|  |  | 236115 | New single family general contractors | Medium | 4338 | 14723 | 7.4 | 28.5 |
|  |  | 236116 | New multifamily general contractors | Medium | 58 | 1022 | 0.5 | 2.0 |
|  |  | 236117 | New housing for-sale builders | Medium | 192 | 1639 | 0.8 | 3.2 |
|  |  | 236118 | Residential remodelers | Medium | 3844 | 11908 | 6.0 | 23.1 |
|  |  | 236210 | Industrial building construction | Medium | 60 | 3537 | 1.8 | 6.8 |
|  |  | 236220 | Commercial building construction | Medium | 986 | 18808 | 9.4 | 36.4 |
|  | 237 |  | Heavy and civil engineering construction |  | 1084 | 20576 | 10.3 | 100.0 |
|  |  | 237110 | Water and sewer system construction | High | 320 | 4205 | 2.1 | 20.4 |
|  |  | 237120 | Oil and gas pipeline construction | High | 37 | 1169 | 0.6 | 5.7 |
|  |  | 237130 | Power and communication system construction | High | 197 | 4195 | 2.1 | 20.4 |
|  |  | 237210 | Land subdivision | High | 109 | 1117 | 0.6 | 5.4 |
|  |  | 237310 | Highway, street, and bridge construction | High | 239 | 6550 | 3.3 | 31.8 |
|  |  | 237990 | Other heavy construction | High | 183 | 3340 | 1.7 | 16.2 |
|  | 238 |  | Specialty trade contractors |  | 16416 | 127573 | 63.9 | 100.0 |
|  |  | 238111 | Residential poured foundation contractors | High | 905 | 4130 | 2.1 | 3.2 |
|  |  | 238112 | Nonresidential poured foundation contractors | High | 95 | 2230 | 1.1 | 1.7 |
|  |  | 238121 | Residential structural steel contractors | High | 30 | 458 | 0.2 | 0.4 |
|  |  | 238122 | Nonresidential structural steel contractors | High | 66 | 1923 | 1.0 | 1.5 |
|  |  | 238131 | Residential framing contractors | High | 748 | 3978 | 2.0 | 3.1 |
|  |  | 238132 | Nonresidential framing contractors | High | 78 | 645 | 0.3 | 0.5 |
|  |  | 238141 | Residential masonry contractors | High | 303 | 860 | 0.4 | 0.7 |
|  |  | 238142 | Nonresidential masonry contractors | High | 65 | 1077 | 0.5 | 0.8 |
|  |  | 238151 | Residential glass and glazing contractors | Medium | 142 | 847 | 0.4 | 0.7 |
|  |  | 238152 | Nonresidential glass and glazing contractors | Medium | 67 | 1403 | 0.7 | 1.1 |
|  |  | 238161 | Residential roofing contractors | High | 847 | 4749 | 2.4 | 3.7 |
|  |  | 238162 | Nonresidential roofing contractors | High | 93 | 2536 | 1.3 | 2.0 |
|  |  | 238171 | Residential siding contractors | High | 515 | 2111 | 1.1 | 1.7 |
|  |  | 238172 | Nonresidential siding contractors | High | 22 | 373 | 0.2 | 0.3 |
|  |  | 238191 | Other residential exterior contractors | High | 123 | 385 | 0.2 | 0.3 |
|  |  | 238192 | Other nonresidential exterior contractors | High | 85 | 786 | 0.4 | 0.6 |
|  |  | 238211 | Residential electrical contractors | Medium | 1470 | 7459 | 3.7 | 5.8 |
|  |  | 238212 | Nonresidential electrical contractors | Medium | 677 | 15418 | 7.7 | 12.1 |
|  |  | 238221 | Residential plumbing and HVAC contractors | Medium | 1600 | 11992 | 6.0 | 9.4 |
|  |  | 238222 | Nonresidential plumbing and HVAC contractors | Medium | 460 | 14076 | 7.0 | 11.0 |
|  |  | 238291 | Other residential equipment contractors | Medium | 61 | 417 | 0.2 | 0.3 |
|  |  | 238292 | Other nonresidential equipment contractors | Medium | 207 | 3231 | 1.6 | 2.5 |
|  |  | 238311 | Residential drywall contractors | Medium | 737 | 6533 | 3.3 | 5.1 |
|  |  | 238312 | Nonresidential drywall contractors | Medium | 115 | 4227 | 2.1 | 3.3 |
|  |  | 238321 | Residential painting contractors | Medium | 1846 | 6281 | 3.1 | 4.9 |
|  |  | 238322 | Nonresidential painting contractors | Medium | 194 | 2589 | 1.3 | 2.0 |
|  |  | 238331 | Residential flooring contractors | Medium | 890 | 1828 | 0.9 | 1.4 |
|  |  | 238332 | Nonresidential flooring contractors | Medium | 72 | 611 | 0.3 | 0.5 |
|  |  | 238341 | Residential tile and terrazzo contractors | Medium | 407 | 1368 | 0.7 | 1.1 |
|  |  | 238342 | Nonresidential tile and terrazzo contractors | Medium | 23 | 165 | 0.1 | 0.1 |
|  |  | 238351 | Residential finish carpentry contractors | Low | 894 | 4065 | 2.0 | 3.2 |
|  |  | 238352 | Nonresidential finish carpentry contractors | Low | 95 | 890 | 0.4 | 0.7 |
|  |  | 238391 | Other residential finishing contractors | Low | 93 | 691 | 0.3 | 0.5 |
|  |  | 238392 | Other nonresidential finishing contractors | Low | 139 | 997 | 0.5 | 0.8 |
|  |  | 238911 | Residential site preparation contractors | High | 1013 | 5172 | 2.6 | 4.1 |
|  |  | 238912 | Nonresidential site preparation contractors | High | 293 | 4777 | 2.4 | 3.7 |
|  |  | 238991 | All other residential trade contractors | Medium | 695 | 2976 | 1.5 | 2.3 |
|  |  | 238992 | All other nonresidential trade contractors | Medium | 256 | 3322 | 1.7 | 2.6 |

**Table S3.** Summary of the number of days that exceeded daily PM_2.5_ concentration thresholds for each WA county with PM_2.5_ data, 2011-2020.

|  |  |  |  | **Days where PM_2.5_ > 20.5 µg/m^3^** | | | | | | | | | | | |
| --- | --- | --- | --- | --- | --- | --- | --- | --- | --- | --- | --- | --- | --- | --- | --- |
| **County** | **N >35 µg/m^3 a^** | **N >20.5 µg/m^3 b^** | **N >55.5 µ/m^3 c^** | **Jan** | **Feb** | **Mar** | **Apr** | **May** | **Jun** | **Jul** | **Aug** | **Sep** | **Oct** | **Nov** | **Dec** |
| Adams | 34 | 73 | 21 | 0 | 0 | 0 | 0 | 0 | 1 | 2 | 46 | 23 | 0 | 1 | 0 |
| Asotin | 54 | 185 | 23 | 10 | 0 | 0 | 0 | 0 | 0 | 2 | 43 | 43 | 28 | 40 | 19 |
| Benton | 29 | 82 | 15 | 7 | 0 | 0 | 0 | 0 | 0 | 0 | 36 | 22 | 1 | 13 | 3 |
| Chelan | 76 | 171 | 45 | 8 | 2 | 0 | 0 | 0 | 0 | 6 | 59 | 45 | 11 | 23 | 17 |
| Clallam | 14 | 23 | 12 | 0 | 0 | 0 | 0 | 0 | 0 | 0 | 14 | 9 | 0 | 0 | 0 |
| Clark | 32 | 122 | 11 | 28 | 6 | 0 | 0 | 0 | 0 | 1 | 16 | 16 | 2 | 27 | 26 |
| Columbia | 25 | 47 | 12 | 0 | 0 | 0 | 0 | 0 | 0 | 0 | 25 | 15 | 0 | 7 | 0 |
| Cowlitz | 16 | 48 | 11 | 4 | 1 | 0 | 0 | 0 | 0 | 0 | 11 | 12 | 0 | 15 | 5 |
| Franklin | 29 | 71 | 14 | 2 | 0 | 0 | 0 | 0 | 0 | 1 | 37 | 22 | 0 | 7 | 2 |
| Garfield | 19 | 39 | 11 | 0 | 0 | 0 | 0 | 0 | 0 | 0 | 22 | 15 | 2 | 0 | 0 |
| Grant | 37 | 93 | 20 | 4 | 0 | 0 | 0 | 0 | 1 | 2 | 49 | 28 | 0 | 6 | 3 |
| Grays Harbor | 12 | 22 | 9 | 0 | 0 | 0 | 0 | 0 | 0 | 0 | 9 | 11 | 1 | 1 | 0 |
| Jefferson | 17 | 23 | 10 | 0 | 1 | 0 | 0 | 0 | 0 | 0 | 12 | 9 | 0 | 1 | 0 |
| King | 26 | 65 | 13 | 5 | 0 | 0 | 0 | 0 | 0 | 3 | 22 | 15 | 5 | 10 | 5 |
| Kitsap | 20 | 44 | 9 | 4 | 0 | 0 | 0 | 0 | 0 | 7 | 16 | 14 | 0 | 3 | 0 |
| Kittitas | 65 | 164 | 24 | 31 | 8 | 0 | 0 | 0 | 0 | 2 | 34 | 40 | 9 | 16 | 24 |
| Klickitat | 18 | 39 | 10 | 0 | 0 | 0 | 0 | 0 | 0 | 0 | 15 | 16 | 0 | 4 | 4 |
| Lewis | 9 | 36 | 5 | 1 | 0 | 0 | 0 | 0 | 0 | 0 | 18 | 3 | 1 | 11 | 2 |
| Mason | 19 | 64 | 10 | 10 | 0 | 0 | 0 | 0 | 0 | 0 | 17 | 15 | 3 | 12 | 7 |
| Okanogan | 76 | 155 | 47 | 6 | 1 | 0 | 1 | 1 | 0 | 8 | 67 | 40 | 0 | 12 | 19 |
| Pend Oreille | 5 | 5 | 5 | 0 | 1 | 1 | 3 | 0 | -- | -- | -- | -- | -- | -- | 0 |
| Pierce | 31 | 135 | 14 | 36 | 2 | 0 | 0 | 0 | 0 | 6 | 19 | 18 | 7 | 22 | 25 |
| San Juan | 0 | 0 | 0 | 0 | 0 | 0 | 0 | 0 | -- | -- | -- | -- | -- | -- | -- |
| Skagit | 5 | 14 | 3 | 0 | 0 | 0 | 0 | 0 | 0 | 1 | 12 | 1 | 0 | 0 | 0 |
| Snohomish | 28 | 140 | 10 | 33 | 5 | 0 | 0 | 0 | 0 | 5 | 22 | 15 | 0 | 29 | 31 |
| Spokane | 53 | 163 | 23 | 14 | 1 | 0 | 0 | 0 | 1 | 6 | 50 | 35 | 4 | 44 | 8 |
| Stevens | 53 | 107 | 22 | 4 | 0 | 0 | 0 | 0 | 0 | 4 | 57 | 23 | 1 | 16 | 2 |
| Thurston | 25 | 115 | 14 | 30 | 3 | 0 | 0 | 0 | 0 | 0 | 16 | 17 | 8 | 24 | 17 |
| Walla Walla | 33 | 80 | 15 | 3 | 0 | 0 | 0 | 0 | 0 | 0 | 34 | 19 | 6 | 12 | 6 |
| Whatcom | 22 | 40 | 10 | 0 | 0 | 0 | 0 | 0 | 0 | 2 | 22 | 13 | 0 | 3 | 0 |
| Whitman | 28 | 60 | 13 | 0 | 0 | 0 | 0 | 0 | 0 | 0 | 39 | 21 | 0 | 0 | 0 |
| Yakima | 70 | 279 | 27 | 47 | 16 | 4 | 0 | 0 | 0 | 6 | 46 | 50 | 12 | 49 | 49 |

^a^ US EPA NAAQS standard

^b^ WA “encouraged” threshold

^c^ WA “required” threshold

**Table S4.** Summary of construction workers, the number of days per year that exceeded PM_2.5_ concentration thresholds, and the estimated demand for respiratory protection based on the 55.5 µg/m^3^ threshold for each WA county in 2020. (Note: construction employment for Garfield County was 2017 due to data availability.)

|  |  |  |  |  | **Days where PM_2.5_ > 55.5 µg/m^3^** | | | | | | | | | | | |  |
| --- | --- | --- | --- | --- | --- | --- | --- | --- | --- | --- | --- | --- | --- | --- | --- | --- | --- |
| **County** | **Workers** | **N >35 µg/m^3 a^** | **N >20.5 µg/m^3 b^** | **N >55.5 µ/m^3 c^** | **Jan** | **Feb** | **Mar** | **Apr** | **May** | **Jun** | **Jul** | **Aug** | **Sep** | **Oct** | **Nov** | **Dec** | **Respirator Demand** |
| Adams | 96 | 8 | 8 | 8 | 0 | 0 | 0 | 0 | 0 | 0 | 0 | 0 | 8 | 0 | 0 | 0 | 768 |
| Asotin | 522 | 12 | 25 | 8 | 0 | 0 | 0 | 0 | 0 | 0 | 0 | 0 | 7 | 0 | 0 | 1 | 4176 |
| Benton | 7359 | 8 | 11 | 8 | 0 | 0 | 0 | 0 | 0 | 0 | 0 | 0 | 8 | 0 | 0 | 0 | 58872 |
| Chelan | 1826 | 12 | 13 | 11 | 0 | 0 | 0 | 0 | 0 | 0 | 0 | 0 | 11 | 0 | 0 | 0 | 20086 |
| Clallam | 1154 | 7 | 7 | 6 | 0 | 0 | 0 | 0 | 0 | 0 | 0 | 0 | 6 | 0 | 0 | 0 | 6924 |
| Clark | 14347 | 8 | 11 | 8 | 0 | 0 | 0 | 0 | 0 | 0 | 0 | 0 | 8 | 0 | 0 | 0 | 114776 |
| Columbia | 130 | 8 | 8 | 8 | 0 | 0 | 0 | 0 | 0 | 0 | 0 | 0 | 8 | 0 | 0 | 0 | 1040 |
| Cowlitz | 2730 | 9 | 10 | 8 | 0 | 0 | 0 | 0 | 0 | 0 | 0 | 0 | 8 | 0 | 0 | 0 | 21840 |
| Douglas | 689 | -- | -- | -- | -- | -- | -- | -- | -- | -- | -- | -- | -- | -- | -- | -- | -- |
| Ferry | 49 | -- | -- | -- | -- | -- | -- | -- | -- | -- | -- | -- | -- | -- | -- | -- | -- |
| Franklin | 2403 | 8 | 9 | 8 | 0 | 0 | 0 | 0 | 0 | 0 | 0 | 0 | 8 | 0 | 0 | 0 | 19224 |
| Garfield | 3 | 9 | 11 | 7 | 0 | 0 | 0 | 0 | 0 | 0 | 0 | 0 | 7 | 0 | 0 | 0 | 21 |
| Grant | 1666 | 10 | 11 | 8 | 0 | 0 | 0 | 0 | 0 | 0 | 0 | 0 | 8 | 0 | 0 | 0 | 13328 |
| Grays Harbor | 1101 | 8 | 9 | 6 | 0 | 0 | 0 | 0 | 0 | 0 | 0 | 0 | 6 | 0 | 0 | 0 | 6606 |
| Island | 1122 | -- | -- | -- | -- | -- | -- | -- | -- | -- | -- | -- | -- | -- | -- | -- | -- |
| Jefferson | 668 | 8 | 8 | 6 | 0 | 0 | 0 | 0 | 0 | 0 | 0 | 0 | 6 | 0 | 0 | 0 | 4008 |
| King | 72075 | 9 | 11 | 7 | 0 | 0 | 0 | 0 | 0 | 0 | 0 | 0 | 7 | 0 | 0 | 0 | 504525 |
| Kitsap | 4585 | 9 | 12 | 6 | 0 | 0 | 0 | 0 | 0 | 0 | 0 | 0 | 6 | 0 | 0 | 0 | 27510 |
| Kittitas | 1085 | 11 | 13 | 7 | 0 | 0 | 0 | 0 | 0 | 0 | 0 | 0 | 7 | 0 | 0 | 0 | 7595 |
| Klickitat | 268 | 9 | 10 | 8 | 0 | 0 | 0 | 0 | 0 | 0 | 0 | 0 | 8 | 0 | -- | -- | 2144 |
| Lewis | 1318 | 0 | 0 | 0 | 0 | 0 | 0 | 0 | 0 | 0 | 0 | 0 | -- | 0 | 0 | 0 | 0 |
| Lincoln | 259 | -- | -- | -- | -- | -- | -- | -- | -- | -- | -- | -- | -- | -- | -- | -- | -- |
| Mason | 645 | 9 | 12 | 7 | 0 | 0 | 0 | 0 | 0 | 0 | 0 | 0 | 7 | 0 | 0 | 0 | 4515 |
| Okanogan | 464 | 11 | 14 | 9 | 0 | 0 | 0 | 0 | 0 | 0 | 0 | 0 | 9 | 0 | 0 | 0 | 4176 |
| Pacific | 277 | -- | -- | -- | -- | -- | -- | -- | -- | -- | -- | -- | -- | -- | -- | -- | -- |
| Pend Oreille | 132 | 0 | 0 | 0 | -- | -- | -- | -- | -- | -- | -- | -- | -- | -- | -- | 0 | 0 |
| Pierce | 23470 | 8 | 13 | 7 | 0 | 0 | 0 | 0 | 0 | 0 | 0 | 0 | 7 | 0 | 0 | 0 | 164290 |
| San Juan | 706 | -- | -- | -- | -- | -- | -- | -- | -- | -- | -- | -- | -- | -- | -- | -- | -- |
| Skagit | 3795 | 0 | 0 | 0 | 0 | 0 | 0 | 0 | 0 | 0 | 0 | -- | 0 | 0 | 0 | 0 | 0 |
| Skamania | 85 | -- | -- | -- | -- | -- | -- | -- | -- | -- | -- | -- | -- | -- | -- | -- | -- |
| Snohomish | 23140 | 9 | 13 | 6 | 0 | 0 | 0 | 0 | 0 | 0 | 0 | 0 | 6 | 0 | 0 | 0 | 138840 |
| Spokane | 12599 | 7 | 11 | 7 | 0 | 0 | 0 | 0 | 0 | 0 | 0 | 0 | 7 | 0 | 0 | 0 | 88193 |
| Stevens | 457 | 9 | 11 | 7 | 0 | 0 | 0 | 0 | 0 | 0 | 0 | 0 | 7 | 0 | 0 | 0 | 3199 |
| Thurston | 6163 | 7 | 12 | 7 | 0 | 0 | 0 | 0 | 0 | 0 | 0 | 0 | 7 | 0 | 0 | 0 | 43141 |
| Wahkiakum | 46 | -- | -- | -- | -- | -- | -- | -- | -- | -- | -- | -- | -- | -- | -- | -- | -- |
| Walla Walla | 929 | 8 | 9 | 8 | 0 | 0 | 0 | 0 | 0 | 0 | 0 | 0 | 8 | 0 | 0 | 0 | 7432 |
| Whatcom | 6754 | 9 | 9 | 7 | 0 | 0 | 0 | 0 | 0 | 0 | 0 | 0 | 7 | 0 | 0 | 0 | 47278 |
| Whitman | 427 | 7 | 8 | 7 | 0 | 0 | 0 | 0 | 0 | 0 | 0 | 0 | 7 | 0 | 0 | 0 | 2989 |
| Yakima | 3729 | 13 | 24 | 8 | 0 | 0 | 0 | 0 | 0 | 0 | 0 | 0 | 8 | 0 | 0 | 0 | 29832 |

^a^ US EPA NAAQS standard

^b^ WA “encouraged” threshold

^c^ WA “required” threshold

**Table S5.** Summary of Pearson correlation between the percent construction workforce and measures of air quality on the monthly timescale for Washington State counties, 2011-2020.

| **County** | **Average AQI days per month** | **Average PM_2.5_ (μg/m^3^)** | **Percent Urban ^a^** | **Percent Rural ^a^** |
| --- | --- | --- | --- | --- |
| Adams | 0.119 | 0.040 | 46.8 | 53.2 |
| Asotin | 0.510 | 0.393 | 94.2 | 5.8 |
| Benton | 0.345 | 0.150 | 87.7 | 12.3 |
| Chelan | 0.468 | 0.215 | 62.0 | 38.0 |
| Clallam | 0.547 | 0.456 | 52.2 | 47.8 |
| Clark | 0.320 | 0.351 | 82.4 | 17.6 |
| Columbia | 0.589 | 0.637 | 67.2 | 32.8 |
| Cowlitz | 0.673 | 0.168 | 67.4 | 32.6 |
| Franklin | 0.462 | 0.429 | 80.2 | 19.8 |
| Garfield | 0.821 | 0.882 | 0.0 | 100.0 |
| Grant | 0.462 | 0.341 | 52.8 | 47.2 |
| Grays Harbor | 0.661 | 0.534 | 60.6 | 39.4 |
| Jefferson | 0.525 | 0.454 | 44.8 | 55.2 |
| King | 0.516 | 0.629 | 96.3 | 3.7 |
| Kitsap | 0.534 | 0.592 | 80.4 | 19.6 |
| Kittitas | 0.283 | 0.263 | 59.5 | 40.5 |
| Klickitat | 0.518 | 0.533 | 41.7 | 58.3 |
| Lewis | 0.445 | 0.264 | 35.9 | 64.1 |
| Mason | 0.657 | 0.118 | 25.3 | 74.7 |
| Okanogan | 0.465 | 0.194 | 21.4 | 78.6 |
| Pend Oreille | -0.401 | 0.246 | 0.0 | 100.0 |
| Pierce | 0.452 | 0.380 | 92.1 | 7.9 |
| San Juan | -- | -0.212 | 0.0 | 100.0 |
| Skagit | 0.332 | 0.459 | 67.1 | 32.9 |
| Snohomish | 0.334 | 0.106 | 89.0 | 11.0 |
| Spokane | 0.508 | 0.501 | 86.1 | 13.9 |
| Stevens | 0.509 | 0.230 | 21.1 | 78.9 |
| Thurston | 0.365 | 0.148 | 75.3 | 24.7 |
| Walla Walla | 0.462 | 0.245 | 81.0 | 19.0 |
| Whatcom | 0.406 | 0.277 | 67.7 | 32.3 |
| Whitman | 0.504 | 0.591 | 67.4 | 32.6 |
| Yakima | 0.196 | 0.109 | 71.2 | 28.8 |

^a^ Percent of county population considered urban or rural calculated from 2000 US Census table P005; <https://data.census.gov/cedsci/table?q=rural%20population&g=0400000US53%240500000&tid=DECENNIALSF32000.P005&hidePreview=true>. Note: the appropriate Summary File was not released in 2010.
